# The p53 Protein Family in the Response of Tumor Cells to Ionizing Radiation: Problem Development

**DOI:** 10.32607/actanaturae.11247

**Published:** 2021

**Authors:** O. A. Kuchur, D. O. Kuzmina, M. S. Dukhinova, A. A. Shtil

**Affiliations:** ITMO University, Saint-Petersburg, 191002 Russia; Blokhin National Medical Research Center of Oncology, Moscow, 115478 Russia

**Keywords:** p53 family protein, cell death, radioresistance, radiation therapy, malignant tumors

## Abstract

Survival mechanisms are activated in tumor cells in response to therapeutic ionizing radiation. This reduces a treatment’s effectiveness. The p53, p63, and p73 proteins belonging to the family of proteins that regulate the numerous pathways of intracellular signal transduction play a key role in the development of radioresistance. This review analyzes the p53-dependent and p53-independent mechanisms involved in overcoming the resistance of tumor cells to radiation exposure

## INTRODUCTION

Ionizing radiation, which ranges from the original use of photons to modern sources of ionizing particles (protons, electrons, neutrons, and carbon atoms), is a key tool in treating tumors. Its effectiveness has been proven for more than 50 years. However, the problem related to the resistance of tumor cells to ionizing radiation (either primary resistance or that acquired during treatment) remains to be solved. Identically to drug resistance, resistance to radiation is an unfavorable prognostic factor of treatment effectiveness. There are numerous reasons why resistance to ionizing radiation develops. This review analyzes the molecular mechanisms forming a synergistic response from tumor cells to radiation therapy with gamma photons. The response needs to cause cell death rather than immune evasion, which may result in cancer cell survival and the formation of a recurrent, radioresistant tumor.

The genotoxic effect (disruption of DNA structure and functions) is considered to be the primary reason why ionizing radiation damages tumor cells. This effect can either be caused by direct rupturing of molecular bonds due to the ionization of atoms in DNA or be an indirect process occurring due to water radiolysis. In the latter case, the interaction between the radiation energy and water molecules gives rise to reactive radicals that cause single- or double-strand DNA breaks. This process can be accompanied by the altering of the expression of the genes whose products are involved in homeostasis regulation [1, 2, 3]. Therefore, the biological effect of radiation is implemented through the regulation of gene transcription. It is plasticity, a shared feature of all living systems that is especially marked in tumor cells, that allows for the rearranging (reprogramming) of the transcription machinery for adaptation to stress. It is quite expected that the transcriptional protein p53, a prototype of the family comprising p63 and p73, is the primary and key sensor regulating the cellular response to radiation-induced DNA damage [4, 5]. The p53-family proteins regulate the cellular response to radiation, thus maintaining the balance between cell survival and apoptosis [6, 7, 8].

The research into the p53 family started in 1979, when independent researchers discovered the protein forming a complex with the known tumor-associated protein, the polyomavirus SV40 large T antigen [9]. The new protein was examined as an auxiliary protein involved in cell malignization by the SV40 virus and expression of small T and large T antigens of the virus in host cells. Back then, serum containing a previously unstudied factor with a molecular weight of 53–54 kDa was also obtained [10]. The era of p53 had arrived: new functions for this protein were being discovered, including such functions as regulation of the cell cycle and the balance between cell survival and death, as well as control over tumor emergence and progression. While previously recognized as a common regulator of cell transformation, p53 and the processes mediated by it have become some of the main topics of discussion in modern molecular oncobiology [11]. The problem remains relevant, as it remains impossible to investigate the novel mechanisms of tumor cell response to ionizing radiation (and largely, the radioresistance mechanisms) without taking into account the significant role played by the p53 family.

Has this problem been solved over the past decades of research? What remains to be clarified in a broad range of questions regarding the role played by the p53 family as the main molecular mechanism in the cell response to ionizing radiation? In this review, we have analyzed the available data on p53-family proteins as regulators (sensors) of therapeutic photons. These mechanisms determine the fate of an irradiated cell: whether it dies or becomes radioresistant.

## THE STRUCTURE AND FUNCTIONS OF p53 FAMILY PROTEINS

The p53 protein (393 a.a.r.) consists of five domains; the key ones are the transcriptional activation domain, the DNA-binding domain, and the tetramerization domain [12, 13]. Expression of the *p53 *gene and the activity of the p53 protein are regulated by diverse stress signals, DNA damage being the main one (but not the only one). After single- or double-strand DNA breaks are induced in cells by radiation, ATM and ATR protein kinases activate the transcriptional competence of p53 via phosphorylation at Ser15 [14, 15].

Two other proteins belonging to this family, p63 and p73, also contain domains similar to those found in p53. All three proteins in homotetrameric form regulate transcription [16, 17]. The p73 protein is activated upon exposure to ionizing radiation, DNA-damaging drugs, and medications that disrupt microtubule dynamics through the pathways regulated by c-Abl tyrosine kinase [18]. In all likelihood, there is cooperation between c-Abl and apoptosis activation by the p73 protein [19]. Much less is known about the features of p63 functions. It has been reported that this protein can also be activated in response to UV and gamma radiation and mediates apoptosis even if p53 is inactivated [20]; upregulated p63 expression in some types of tumors reduces cellular sensitivity to ionizing radiation [21]. Since there is a high level of structural similarity between the proteins belonging to this family, full-length p73 and p63 are capable of binding and activating the transcription of most of the p53-dependent promoters [22].

## MUTATIONS AND ISOFORMS OF p53-FAMILY PROTEINS IN TUMOR CELLS

**Fig. 1:**
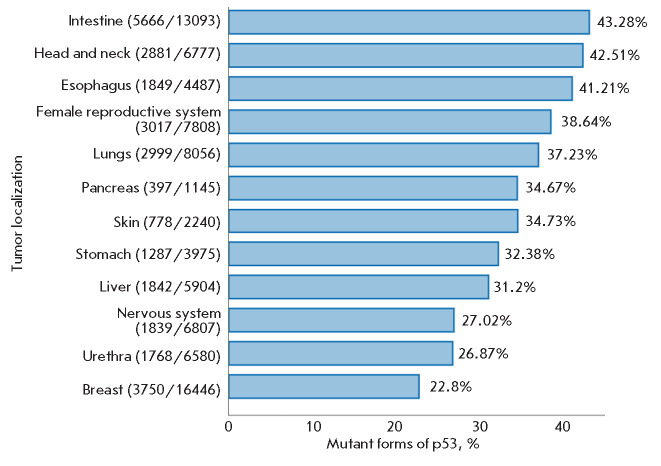
Prevalence of mutant p53 forms in tumors based on DNA sequencing (IARCTP53 Database, 2019). X-axis: the number of biopsy specimens with identified mutations; Y-axis: the number of analyzed biopsy specimens

The disruption of the functions of p53-family proteins can be caused by mutations in the *TP53*, *TP63*, and* TP73 *genes or the genes whose products are involved in the modification of these proteins (e.g., protein kinases phosphorylating p53 (Cdc2, JNK1, protein kinase C)) [23]. The *p53 *gene encodes nine protein isoforms (p53, p53β, p53γ, Δ133p53, Δ133p53β, Δ133p53γ, Δ40p53, Δ40p53β, and Δ40p53γ); this diversity is determined by alternative mRNA splicing, alternative use of the promoter, or translation initiation sites [24]. An analysis of the biopsy specimens of 29,346 tumors derived from different tissues showed that most of these tumors carry a mutant p53 (*Fig. 1*). Most malfunctions of p53 in tumor cells are caused by missense and/or point mutations; there can also be deletions and splicing errors [25]. Approximately 15% of the mutations in the* p53 *gene are frameshift or nonsense mutations [26]. In most tumors, *TP53 *mutations are found in exons 5–8 encoding the DNA-binding domain. Because of this, 80% of missense p53 mutations are associated with the pro-oncogenic function [27, 28].

The main difference between most mutant forms of p53 and wild-type p53 (whose half-life in dormant cells does not exceed 5–10 min) consists in enhanced stability because of the disrupted negative feedback with E3 ligase Mdm2 and binding to Hsp90 and Hsc70, which stabilizes p53 and causes its accumulation in cells [29, 30]. Importantly, mutant p53 can form oligomeric complexes with wild-type p53. This binding can inactivate the intact protein and explains why mutant p53 can transform cells in the presence of wild-type protein [31].

A wide range of the isoforms of two other proteins belonging to the p53 family are known: the *p63 *and* p73 *genes contain an internal promoter in intron 3 and, due to alternative splicing, express the 6 and 35 mRNA variants, respectively. The *p63 *gene is located in the 3q27-ter locus; three C-terminal isoforms (α, β, and γ) formed as a result of alternative splicing are expressed from it. The *p73 *gene is located in the 1p36 locus; its alternatively spliced transcripts encode the C-terminal isoforms α–η [32]. *p63 *and *p73 *mRNA can be transcribed from the distal and internal (in intron 3) promoters. The distal promoter regulates *TAp63 *and* TAp73 *expression (the transactivation domains are homologous to *p53*), whereas the ΔNp63 and ΔNp73 isoforms, which are N-terminal truncated proteins (ΔN) with properties in opposition to those of the p63/ TAp73 isoforms, are transcribed from the internal promoter [33]. These results indicate that the p53 family is exceptionally diverse. It is little surprise that the problem under examination remains relevant while also acquiring new layers of complexity.

## RESPONSE TO THERAPEUTIC IONIZING RADIATION

**The p53 protein**

**Fig. 2:**
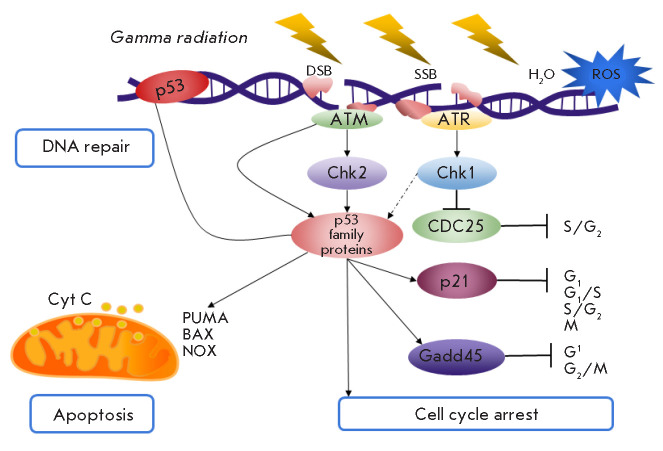
The response mechanisms to ionizing radiation involving p53-family proteins

As mentioned above, p53 is activated in response to stressful conditions (primarily, to DNA damage caused by oxidative stress, ionizing radiation, etc.) The proteins activating the protein kinases ATM (ataxia telangiectasia mutated kinase) and ATR (ATM- and Rad3-related kinase) bind to the DNA damage site [34]. In turn, the latter group of proteins activates the checkpoint kinases Chk1 and Chk2 phosphorylating p53 at Ser15. Activation of p53 results in the induction of Mdm2, its functional antagonist. Binding between Mdm2 and the N-terminus of p53 promotes monoubiquitination of p53 and nuclear export or polyubiquitination and p53 hydrolysis in the proteasome [35, 36]. *Figure 2* shows a generalized scheme of the intracellular responses to ionizing radiation involving p53-family proteins.

The “choice” between cell survival and death is regulated by post-translational modifications of p53 and its isoforms, partner proteins, and a set of activated genes [37]. The p53 protein activates the transcription of p21Cip1/Waf1, blocker of the cell cycle at the G1 phase that inhibits binding of cyclins A and B to CDK1 and CDK2 protein kinases [38, 39]. There is insufficient data on the role played by p53 in the regulation of the S phase of the cell cycle. During the S phase, Chk2 phosphorylates phosphatase CDC25A, causing its degradation and cell cycle arrest [40]. The p53 protein can delay the G2/M progression through repression of *CDC2 *and cyclin B promoters [41].

In response to radiation, p53 can stimulate apoptosis through the induction of proapoptotic (Bax) and repression of antiapoptotic (Bcl-2) proteins, as well as the activation or inhibition of the other target genes involved in cell cycle regulation. It is known that low-dose radiation induces p21 and Hdm2 (an Mdm2 homolog), while high-dose radiation increases the Bax : Bcl-2 ratio, thus promoting apoptosis [42]. Radioresistance is caused by the activity of antiapoptotic proteins (overexpression of the Bcl-2 family proteins), loss of the components of apoptotic cell signaling, or inhibition of the genes encoding caspases.

The efficiency of DNA damage repair in response to radiation depends on the histological origin of the cells and cell cycle phase. The G2 and mitotic phases are most sensitive to it. Importantly, p53 may play a dual role in response to radiation exposure. In some cases, an increased p53 expression level enhances sensitivity to radiation, while correlation between an increased p53 expression level and radioresistance has been demonstrated in other cases [43]. Under minor stress, p53 can act as a survival factor, since it promotes DNA damage repair; therefore, *p53 *knockout in a colon adenocarcinoma cell line (HCT116) increases the sensitivity of cells to radiation and causes the “mitotic catastrophe,” the aberrant chromosome segregation resulting in cell death. A significantly increased number of cells undergoing mitotic catastrophe was also observed in irradiated human fibrosarcoma cells (HT1080) after p53 was inactivated by a dominant negative mutant [44].

The transcription factors Slug and Snail regulate the epithelial–mesenchymal transition (EMT) and invasion by tumor cells of the subjacent tissues [45]. A research team from Seoul National University found that p53 induces Slug and Snail degradation by Mdm2-mediated ubiquitination [46]. Importantly, Snail activity depends on the p53 status. Thus, the mutant forms of p53 cause overexpression of Snail and Slug, which is related to the acquisition of radioresistance by ovarian cancer cells: these proteins increase the survivability of precursor cells thanks to the activation of the SCF/c-Kit signaling pathway [47].

Polo-like serine/threonine protein kinase 3 (PLK3) is one of the components of p53-mediated regulatory signals. PLK3 interacts with p53, Chk2, and CDC25C in response to DNA damage. p53 can bind to *PLK3* promoter and induce expression of its gene, which is followed by a delay in G2/M progression and cell cycle arrest. Another p53-regulated gene, *GPX1*, encodes the antioxidant protein glutathione peroxidase. After irradiation, cells accumulate highly active oxygen free radicals. Due to *GPX1 *induction and rapid catabolism of H_2_O_2_, p53 can protect cells against the oxidative damage that accompanies radiation treatment [48, 49]. The dual role of p53 upon radiation exposure manifests itself here: this protein protects cells in some cases, while in other cases it promotes their death.

Halacli *et al*. revealed that in colon adenocarcinoma cells with non-functional p53, telomerase activity drops after irradiation, while it increases in the wild-type isogenic line (p53+/+). An opposite effect was observed for the catalytic subunit of telomerase (TERT). After irradiation, TERT activity decreases as p53 induction increases, while TERT activity in p53−/− cells is increases. Whereas irradiation does not alter telomerase activity, accelerated senescence is observed in cells with normally functioning p53. Therefore, telomerase activity and G1-phase arrest of cell cycle progression in irradiated cells are regulated depending on the p53 status [50].

The equally important features of cell cycle regulation have been demonstrated for connective tissue cells. Thus, mouse embryonic fibroblasts (MEF p53+/+) accumulated in the G1 phase after irradiation (5 Gy): the p53-dependent promoter of the *p21 *gene was activated in them. However, irradiated p53 knockout cells did not undergo apoptosis and remained in the premitotic phase [51]. In p53−/− cells, p21 and Cdc25 regulated p53-independent cell cycle arrest at the G2 phase [52].

**The p63 and p73 proteins**

The role played by p73 in the cellular response to ionizing radiation has been studied more thoroughly compared to that of p63. It was found that *p73 *expression level is higher in patients with radiosensitive cervical cancer compared to that in patients with radioresistant cervical cancer. The p73 protein is a positive regulator of *p21 *transcription upon irradiation and can potentially take on the role of p53 protein in the regulation of cell cycle checkpoints. Hence, p73 is involved in the regulation of radiosensitivity [53].

Increased p73 expression induced by radiation activates the transcription of the p53-dependent genes* Bax*, *Mdm2*, and *GADD45*, thus promoting apoptosis or cell cycle arrest and inhibiting proliferation. It has been assumed that p73 can be induced by irradiation and take on some of the functions of p53 in tumor cells with disrupted p53 expression or activity. Furthermore, activation of p53 suppresses p73 expression in irradiated breast and lung cancer cells [54, 55, 56]. It has been shown recently that nutlin, a low-molecular-weight agent uncoupling the p53-Mdm2 interaction, can induce apoptosis in p53-negative cells through activation of p73 upon irradiation. These results justify the use of nutlin for treating tumors with non-functional p53 [57].

The antitumor drug cisplatin and ionizing radiation cause Tyr99 phosphorylation of p73 and the accumulation of this protein. This post-translational modification occurs due to the interaction between p73 and tyrosine kinase Abl; it promotes the apoptotic activity of p73. Furthermore, it has been demonstrated that treatment with cisplatin can result in the acetylation of p73 by the p300 protein. These data attest to the importance of p73 in cellular response to a combination of chemotherapy and radiation therapy [58].

A genome-wide association study (GWAS) in p63 and p73 knockout cells has shown that these proteins regulate the transcription of the *BRCA2*, *Rad51*, *Rad50*, and *Mre11 *genes, whose products are involved in the repair of single- and double-strand DNA breaks. This mechanism can be responsible for tumor survival. Interestingly, the ΔNp63 and ΔNp73 isoforms are stronger transactivators of the aforelisted genes than the TA isoforms. An analysis of the mutations in the *p63/p73* genes can be important in choosing a radiation therapy strategy [59].

Therefore, the mutant p53-family forms are regulated through numerous pathways, which are far from obvious in some cases. Proteins belonging to this family mediate the signaling cascades that regulate the establishment of stable phenotypes or death of irradiated cells. The use of platinum-based drugs in combination with mTOR inhibitors or other intracellular signal blockers opens up the potential for modulating p53-family proteins and enhancing the response to ionizing radiation.

## RADIORESISTANCE MEDIATED BY p53-FAMILY PROTEINS

A pioneer study focused on the role played by p53 in the radioresistance of tumor cells was the paper by Lee and Bernstein [60], who used transgenic mice carrying p53^Pro193^ and p53Val135 mutations and showed that the expression of both mutant variants of the* p53 *gene significantly increases the gamma radiation resistance of hematopoietic cells. They uncovered an association between mutations in the p53 gene and radioresistance [60]. The radiosensitivity of rat embryonic fibroblasts (REF) transfected with a mutant form of* p53 *(*MTp53^Pro193^*), either individually or in combination with *H-Ras *and *E7 *oncogenes, was studied later. The results of the experiments involving transfection with* p53^Pro193^* have confirmed the previous data showing that radioresistance of cells increases. Cotransfection with the mutant *p53 *and *H-Ras *genes or transfection with *p53^Pro193^*, *H-Ras *and *E7 *yielded clones with an even higher radioresistance and overexpression of mutant p53 [61].

The ovarian adenocarcinoma cell lines SKOV-3 and CaOV-3 acquired radioresistance if the mutant p53 was overexpressed; irradiation caused neither activation nor accumulation of the mutant p53 form. It turned out that p-53-regulated expression of *Bcl-2 *in these cell lines was associated with gamma radiation resistance and cisplatin sensitivity. It is possible that mutations in the *p53 *gene causing the increased protein expression level and radioresistance are associated with greater p53 stability and cell cycle blockage; cells have time to repair DNA damage [62].

It has been shown for melanoma cells that the Chk2/ hCds1-independent signaling pathway of DNA damage dephosphorylating Ser376 in the C-terminal region of p53 enhances p53 activity upon irradiation. In cells with functional p53, Ser376 phosphorylation is not regulated by DNA damage: so, these cells do not develop radioresistance. Contrariwise, the defects in the superjacent mechanisms of p53 activation in response to DNA damage (e.g., mutations in Chk2/hCds1 disabling Ser376 phosphorylation of p53 upon irradiation) are associated with the development of radioresistance by melanoma cells. The same feature was also observed for the mutant p53, which was unable to interact with the 14-3-3 protein [43].

In cooperation with p53, the Ki-67 nuclear protein, which is expressed in proliferating cells and is non-functional in dormant (G0) cells, is also a predictor of radioresistance. In specimens of head and neck squamous cell carcinoma, the p53 expression level correlates with the absence of a tumor response to radiation therapy. A combination of p53 accumulation and low Ki-67 level is associated with tumor recurrence in patients with early-stage cancer. Therefore, p53 and Ki-67 can play a key role in the choice of radiation therapy strategies for patients with head and neck tumors [63]. Multiple mutations, including changes in p53-dependent proapoptotic proteins Bcl-2, PUMA, and Bax, increase resistance to radiation therapy and chemotherapy [64].

The activity of focal adhesion kinase (FAK) is increased in patients with various tumors. In the FAK knockout cell line of squamous cell carcinoma of the skin, radiation suppresses transcription of the *p21* gene and other p53 target genes mediating cell cycle arrest and DNA damage repair. Suppression of *p53* and *p21 *activation promotes radiosensitization of tumor cells; this was not observed for intact FAK [65]. The experiments on FAK inhibition in p53-negative lung cancer cells showed encouraging results: *in vitro* migration and invasion were reduced, and *in vivo *survivability tended to increase [66]. Modulation of FAK activity, in combination with radiation, seems quite promising.

Overexpression and the accumulation of p53 in endometrial cancer cells are caused by the fact (among others) that mutant p53 is refractory to ubiquitin-mediated proteasomal degradation. Simultaneous accumulation of p53 and PTEN phosphatase renders endometrial cells insensitive to radiation therapy, which is associated with disease progression [67].

Since ionizing radiation induces oxidative stress [68], reactive oxygen species (ROS) are involved in radiation damage to mitochondria. Activation of mitochondrial BNIP3, a proapoptotic protein belonging to the Bcl-2 family and regulating the generation of ROS in irradiated cells and mitophagy, did not take place in the cells with non-functional p53. Thus, p53 acts as a key mechanism in the regulation of BNIP3; the absence of functional p53 can affect the survivability of irradiated tumor cells by maintaining mitochondrial integrity [69]. The p53 status turns out to be an important biomarker for predicting the therapeutic value of drugs targeted at mitochondrial proteins.

There are insufficient data on the role played by p63 and p73 in the formation of radioresistance phenotypes. Since the proteins belonging to this family are interchangeable or complement each other in some cases, it is fair to assume that p63 and p73 can also regulate radioresistance via mechanisms similar to those employed by p53. Indeed, Moergel *et al*. [21] studied p63 in specimens of oral squamous cell carcinoma. The expression level of the transactivated form TAp63 before treatment is a marker of radioresistance; the high levels of TAp63 expression are associated with poor treatment effectiveness and unfavorable prognosis [70, 71]. These results were confirmed by studies of biopsy specimens of squamous cell carcinoma of the head and neck collected from 33 patients; the increased level of p63 expression before treatment in these tumors is also considered a predictor of radioresistance, but studies involving a larger patient cohort are needed [21].

Expression of the ΔNp63α isoform upon irradiation for the cell lines of squamous cell carcinoma of the larynx, head, and neck (PCI-I-1, PCI-13, SCC-68, and SCC-4), as well as primary oral mucosal keratinocytes, has also been studied. The level of ΔNp63α expression was dependent on the radiation dose in all the cell lines. ΔNp63 knockdown induced by small interfering RNA (siRNA) increased radiation sensitivity [72]. However, an opposite effect was also observed: expression of TAp73 and caspase 7 in colorectal cancer cells after radiation therapy correlated with radiosensitivity. The* Rb1 *gene was then knocked down using microRNA miR-622. Rb1 knockdown inhibited the formation of the Rb-E2F1-P/CAF complex, thus reducing the expression of *TAp73 *and caspase 7, and the cells acquired radioresistance [73].

It is also known that upon the irradiation of cells, p63/ p73 bind to the mutant form of p53 in some cases and cannot activate the proapoptotic genes: so, the cells survive. Inhibitors of mutant p53 forms, p63/p73 overexpression, or disruption of physical interactions between proteins belonging to this family using peptidomimetics or low-molecular-weight compounds (see text below) are used to enhance p63/p73 activity [74, 75].

## WAYS TO OVERCOME RADIORESISTANCE UPON MODULATION OF p53-FAMILY PROTEINS

**Modulation of p53**

**Fig. 3:**
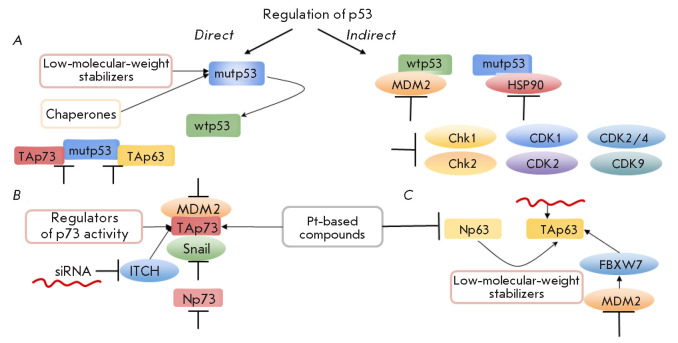
Methods for enhancing the sensitivity of tumor cells to ionizing radiation by modulating the p53-family proteins. (*A*) – Modulation of p53 by low-molecular-weight-stabilizing molecules and chaperones. (*B*) – Regulation of p73 by acting on Snail family proteins and E3 ubiquitin ligase (MDM2, ITCH). (*C*) – The impact on p63 isoforms via Pt-containing compounds, low-molecular-weight stabilizers and ubiquitin ligase activity (MDM2, FBXW7). See explanation in the text

The key approaches to modulating p53 for the radiosensitizing effect include (*Fig. 3*):

1. Low-molecular-weight p53 stabilizers [76];

2. Modulators of chaperones/stabilizers of wild-type and mutant p53 [77];

3. Regulators of E3 ubiquitin ligases;

4. Modulators of components of the p53 signaling pathway (e.g., CDK and Bcl-2) [78].

Stictic acid, which restores the functions of p53 by binding to its mutant form, is one of the examples of low-molecular-weight stabilizers [79]. Carbazole-based compounds also exhibit a similar effect. Thus, PK083 binds to the mutant form p53Y220C and restores its transcriptional activity, causing apoptosis [36, 80, 81]. Analogs of quinazoline (2-styryl-4-aminoquiazoline, CP-31398) [82, 83, 84] reactivate p53. Alkylating agents are involved in the restoration of the structure of the p53 protein by directly binding to and modifying its mutant forms [85]. PRIMA-1 and its more efficient analog, PRIMA-1Met (APR-246), are among such agents that restore p53. Inside the cells, these agents are converted into an active compound, methylene quinuclidinone (MQ), a Michael acceptor that binds covalently to cysteine residues in the DNA-binding domain of p53. Cys277 is essential for the MQ-mediated thermal stabilization of the mutant p53R273H, while Cys124 is needed for APR-246-mediated functional restoration of the mutant p53R175H in tumor cells and the normalizing activity of the wild-type protein. These studies are especially important for a rational design of p53-targeting molecules [86, 87, 88].

The activity of p53 can also be regulated indirectly, through stabilizers of the intact or mutant forms of p53. Blanden *et al*. [89, 90] showed that the low-molecular- weight compound ZMC1 (NSC319726) acts as a metallochaperone and restores the functions of p53R175H [89, 90]. In the case of the stabilization of mutant pro-oncogenic forms of p53 by Hsp90, the activity of this chaperone needs to be suppressed in order to sensitize the cell to chemotherapy and radiation therapy. Hsp90 inhibitors (ganetespib and geldanamycin) are used for this purpose, which allows one to suppress the proliferation of tumor cells carrying mutant p53. AUY922 and other candidate drugs destabilize the mutant protein by suppressing the chaperone activity [91, 92, 93, 94]. Cerivastatin, one of the members of the class of statins, inhibits the mevalonate pathway. By inhibiting HMG-CoA reductase (an enzyme catalyzing the synthesis of mevalonic acid), this compound reduces the activity of histone deacetylase HDAC6, resulting in dissociation of the Hsp90–utant p53 complex [95]. Therefore, it is reasonable to assume that destabilization of mutant p53 and restoration of p53 functions can increase cell sensitivity to radiation.

Agents that regulate the interaction between E3 ligases and p53 are being designed. Among the numerous agents uncoupling the Mdm2-p53 interaction, the family of *cis*-imidazolines (nutlins) is universally recognized. AMG-232 is currently undergoing clinical trials [96]. Anthraquinones activating p53 via Mdm2 suppression also possess a high therapeutic potential [97, 98]. There is a diverse range of Mdm2 inhibitors: genisteins, curcumins, ginsenosides, SP141, and NFAT1-Mdm2 dual inhibitors. Thus, curcumin, a natural compound exhibiting antioxidant properties, can stabilize p53 by forming a stable complex between p53 and (NAD(P)H:quinone oxidoreductase 1 [99], while genistein can amplify cell death through p53-dependent apoptosis [100, 101, 102]. Ma *et al*. [103] investigated USP14, a signalosome COPS5 activator enhancing the activity of E3 ligase, as a potentially promising target for therapy and endeavored to choose inhibitors for it (e.g., IU1 and AP15).

Modulation of p53 can occur indirectly via the regulation of the components of the p53 signaling pathway. One of the promising strategies can involve affecting cyclin-dependent kinases, which regulate the cell cycle and transcription [104]. Treatment with roscovitine, a CDK1 and CDK2 inhibitor, has induced the apoptosis of cells expressing mutant p53 [105, 106]. Chemical inhibitors of mTOR (mammalian target of rapamycin), the cyclin-dependent protein kinases CDK1, CDK7, and CDK9, as well as poly(ADP-riboso)polymerases (PARP), also affect p53 functions. Roscovitine and flavopiridol increase the p53 expression level in cells and reduce *Mdm2 *transcription, possibly by inhibiting CDK7 or CDK9, which are components of the general transcription machinery [107]. The effect of CDK inhibitors flavopiridol, THZ1 and YKL-1-116 on *Mdm2* transcription and p53 induction was studied using an Mdm2:T2A-GFP reporter; its transactivation in breast cancer cells (MCF-7 cell line) was quantified. Flavopiridol and roscovitine increased p53 transactivation as a result of Mdm2 depletion. Although p53 is probably inactive in these situations (since transcription in the presence of an inhibitor of transcriptional protein kinases is either disrupted or absent), after CDK7 and CDK9 inhibitors (THZ1 and YKL-1-116, respectively) are removed, p53 activates the targets (DR5, Fas and p21) and enhances the antitumor effect of irradiation [108, 109].

**Fig. 4:**
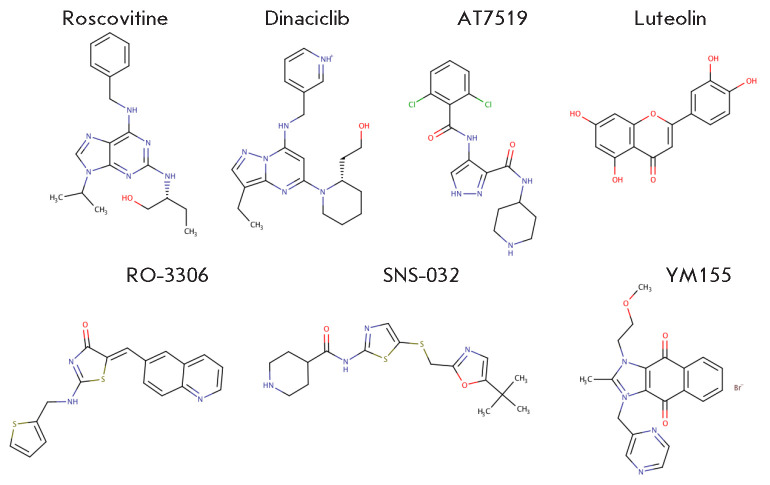
Therapeutically promising inhibitors of the cyclin-dependent protein kinases modulating the activity of p53

CDK2, CDK5, CDK9, and CDK12) also resulted in a switch to p53-dependent apoptosis [110, 111]. Furthermore, AT7519 (an inhibitor of CDK1, CDK2, CDK4, CDK6, and CDK9) and SNS-032 (an inhibitor of CDK2, CDK7, and CDK9) increases sensitivity to irradiation through p53 activation and Chk1 suppression [112]. Compound YM155 affects the cell cycle regulation through Chk1 and Chk2 by stabilizing p53 and p21 [113]. The thiazole derivative of quinone RO-3306, an inhibitor of CCNB1/Cdk1, induces p53-mediated apoptosis of p53-intact neuroblastoma cells [114]. Luteolin, which causes Mdm2 degradation, can inhibit cyclin D1 and CDK2/4, thus increasing the level of p53 expression in the cell [115]. Therefore, it is promising to use a combination of CDK inhibitors and radiation therapy. *Figure 4* shows the chemical formulas of CDK inhibitors listed above.

Gene therapeutics and synergistic impacts on cellular metabolism, which may restore or evade the disrupted functions of mutant p53 through the regulation of the metabolism of tumor cells, are also used for tumor treatment, along with chemotherapeutic agents. In the cells with intact p53, ATP is synthesized via oxidative phosphorylation. The loss of normal functions by p53 leaves the cell relying on glycolysis; cells become able to survive under hypoxic conditions. Recent findings indicate that treatment with a glycolysis inhibitor can increase the sensitivity of the tumor to radiation therapy [116].

**Alteration of p73 and p63 activity**

Sensitivity to chemotherapy and radiation therapy can be increased by impacting p53 and other p53-family proteins. Thus, some chemotherapy regimens increase the expression level of p73 [117]. Platinum-based drugs (cisplatin, oxaliplatin, etc.) help the cell overcome drug resistance by increasing activity of the TAp73 protein and inducing the apoptosis of tumor cells [118]. In addition, cisplatin suppresses the pro-oncogenic form ΔNp63α, which can also inhibit tumor growth [119, 120] and presumably enhance its radiosensitivity.

These p53-like strategies can also be applied to p73 and p63 (to regulate the activity of E3 ligases). E3 ligase ITCH negatively regulates p73; ITCH knockout using a combination of nanoparticles and siRNA enhances the stabilization of p73 in p53-mutant cells [121]. Agents directly regulating p53 activity can also be effective in the case of p63 and p73. Curcumin, a p53 stabilizer, activates *p73 *expression [99, 122].

By activating the AMP-activated protein kinase (AMPK), metformin affects all three p53-family proteins: it increases the expression level of p53 and p73, while reducing the expression level of the pro-oncogenic form of p63 (ΔNp63α) [123, 124]. Prodigiosin has a positive effect on *p53 *expression by activating its reporter via induction of p73 and reduction of the expression level of oncogenic ΔNp73, a suppressor of the *p53 *gene [125]. Compound NSC59984 destabilizes the mutant p53 and causes its degradation, which is accompanied by induction of p73-dependent apoptosis [126].

Along with the regulators affecting all proteins belonging to the p53 family, agents with selectivity to individual proteins have also been proposed. *Abrus agglutinin *(AGG), a plant-derived lectin inhibiting translation, leads to p73 induction [127]. The p73 induced by lectin suppresses the expression of Snail and inhibits the EMT in the cells of squamous cell carcinoma of the larynx. It is noteworthy that AGG promotes Snail transfer from the nucleus into the cytoplasm and induces its degradation via ubiquitination. Therefore, AGG stimulates p73 and suppresses the EGF-induced EMT and invasiveness by inhibiting the ERK/Snail pathway [128]. Protoporphyrin IX (PpIX), a metabolite of aminolevulinic acid, which is used in photodynamic cancer therapy, stabilizes TAp73 and activates TAp73-dependent apoptosis in tumor cells lacking p53. TAp73 is activated through the disruption of TAp73/ MDM2 and TAp73/MDMX interactions, as well as the inhibition of TAp73 degradation by ubiquitin ligase ITCH [129]. Similar properties were also observed for 1-carbaldehyde-3,4-dimethoxyxantone, which stabilizes TAp73 by inhibiting its binding to Mdm2 [130]. Diallyl disulfide (DADS) enhances the sensitivity to ionizing radiation by increasing the expression level of TAp73 and reducing the expression level of the ΔNp73 isoform. The DADS-mediated balance between TAp73 and ΔNp73 is associated with the radiosensitivity of cervical cancer cells [131].

The results of the use of microRNA for p63 modulation have been published [132]. miR-130b activates the antitumor p63 isoform (TAp63) by binding directly to the protein [133]. Special attention should be paid to the study of the response of p63 to irradiation and the acquisition of p63-mediated radioresistance, as well as the choice of drugs targeted at a respective gene/protein for designing novel therapy methods, especially for patients with cross-resistance to chemotherapeutics.

The important problem related to the design of methods for targeted drug delivery using liposomes and nanoparticles remains poorly studied. The mesoporous nanoparticles UCNPs(BTZ)@ mSiO_2_-H2A/p53, which contain the proteasome inhibitor bortezomib along with cDNA of p53, increased cell sensitivity to this drug and induced a more pronounced apoptosis compared to the situation in the control cells without nanoparticles in [134]. Not only gene fragments, but also antagonists of E3 ligases for p53 (Mdm2 and MdmX) can be delivered inside cells as a part of gold nanoparticles. Furthermore, the low-molecular-weight agents VIP116 and PM2 inhibiting the p53-Mdm2 and p53-Mdm4 interactions, which were delivered inside lipodisks (the nanosized bilayer structures stabilized into flat circular shapes by lipids linked to polyethylene glycol), significantly reduced the viability of tumor cells [136]. This approach can be used to precipitate the death of tumor cells exposed to ionizing radiation.

## CONCLUSIONS: THE NEW APPROACHES TO AN OLD PROBLEM

Despite the many decades of research, the role played by the p53 protein as a molecular target and a prognostic marker in radiation therapy remains controversial. The situation is complicated by the variability of the p53-dependent responses elicited by the radiation treatment of different tumors (even cell lines originating from the same tissue) [137]. Nonetheless, the p53 protein was reported to be an informative, predictive genetic marker of acute toxicity or response to the radiation therapy of native tumors [138]. By analyzing the expression of p53 and a number of other genes, researchers have predicted the absorbed dose at which a particular tumor response is elicited [139, 140]. Gendicine (Ad-*p53*), a recombinant adenovirus engineered to express wild-type p53 in the tumor where this protein is mutated, can be considered a successful application of p53-targeting therapy. Ad-*p53 *is used in clinical practice and shows a good result when combined with radiation therapy, especially in patients with breast, pancreatic, cervical, or ovarian cancer [141].

Information regarding the application of p63 and p73 in radiation oncology remains so far confined to experimental data and the hypothesis on their practical use [142]. This gap needs filling, since a general analysis of the p53-protein family reveals a more detailed, and more complex, mechanism of radiation response regulation.

The problem related to p53-negative tumors remains unsolved. One of the pathways that allow one to evade the non-functional p53-dependent mechanism is to use nanostructured silver particles that can induce mitochondrial stress and apoptosis independently of p53 [143]. The question of whether these materials can be combined with radiation therapy remains to be elucidated[ 144]. Finally, the impact on p63 and p73 should be considered justified if their functions are preserved in p53-negative tumors [145].

## Abbreviations

EMT: epithelial mesenchymal transition
NF-κB: nuclear factor kappa-light-chain-enhancer of activated B cells
CDK: cyclin-dependent kinases
TAp63/73: p63/p73 isoforms with N-terminal transactivation domain (TA)
ΔNp63/73: p63/p73 isoform with N-terminal transactivation domain deletion
